# Delayed onset arginine vasopressin deficiency after traumatic brain injury

**DOI:** 10.1530/EDM-24-0039

**Published:** 2024-10-28

**Authors:** Silviu-Andrei Tomulescu, José Boto, Karim Gariani

**Affiliations:** 1 Division of General Internal Medicine, Department of Medical Specialties, Geneva University Hospitals, Geneva, Switzerland; 2 Division of Diagnostics, Neuroradiology Department, Geneva University Hospital, Geneva, Switzerland; 3 Division of Endocrinology, Diabetes, Nutrition and Therapeutic Patient Education, Department of Medical Specialties, Geneva University Hospitals, Geneva, Switzerland

**Keywords:** delayed onset, Arginine vasopressin, Traumatic Brain Injury, Neuroendocrine Dysfunction

## Abstract

**Summary:**

Delayed arginine vasopressin deficiency (AVP-D) can present in patients following traumatic brain injury (TBI) and may occur years after the trauma, presenting with nonspecific symptoms. The objective of this case is to highlight the importance of considering the delayed onset AVP-D in patients with a history of TBI. We report a case of a patient who had sustained severe traumatic brain injury 8 years before and who presented with polydipsia, behavioural disorder and frequent falls during the last 3 months. The diagnosis of AVP-D was confirmed by water restriction with a positive response to desmopressin, and pituitary MRI showed an absent spontaneous posterior hyperintensity on T1WI. Follow-up confirmed permanent diabetes insipidus as well as a suspected anterior pituitary deficiency. Pituitary dysfunction occurs following TBI and is correlated with severity. As in our case, symptoms are generally non-specific and are difficult to explore given the patient’s neurologic sequelae. MRI 8 years post trauma showed changes in pituitary morphology. Some authors have proposed the need for active screening of post-TBI patients. This case highlights the need for clinicians to be aware that AVP-D can occur years after traumatic brain injury.

**Learning points:**

- Delayed onset post-traumatic arginine vasopressin deficiency may occur in patients with TBI and is correlated with the severity.
- The clinical picture is usually non-specific and diagnosis of AVP-D is challenging in non-verbal patients.
- An active screening for pituitary dysfunction is warranted in TBI patients and should be extended should one hormone deficit be identified.

## Background

Pituitary hormone abnormalities were reported in about 20–40% of survivors of traumatic brain injury (TBI) with arginine vasopressin deficiency (AVP-D) being the least frequent (1). Seventy-eight percent of patients present post-traumatic hypopituitarism (PTHP) during the first 2 weeks following trauma (2), while the proportion of patients presenting with enduring pituitary dysfunction is more variable and is estimated to be between 5.4% and 76.4% (3). AVP-D occurs in 16–28% of patients with TBI (4) in the acute phase and is positively correlated with the severity of the trauma (4), with 6.9% of patients presenting AVP-D during follow-up studies (4). The typical timeframe for post-traumatic hypopituitarism is within 1 year, but there are reports of a delayed diagnosis of 5 years or more in up to 11.5% of AVP-D patients (5).

The physiopathology of the delayed onset AVP-D is unclear and several hypotheses are assumed. Impairment of the vessels supplying the pituitary gland is one explanation for anterior pituitary deficiency (6, 7). Diffuse compression due to rising intracranial pressure explains both anterior and posterior dysfunction (6, 7). Severe hypovolemia or hypotension such as in Sheehan syndrome, when combined with increased ICP, can also increase the odds of pituitary necrosis (8). Axonal brain injury is another mechanism which would explain posterior pituitary dysfunction (9). For symptoms to occur, 90% of the anti-diuretic hormone (ADH)-secreting neurons extending from the hypothalamus to the posterior pituitary would have to be impaired (10). Finally, the autoimmune hypothesis is the most recent one with the presence of autoantibodies against pituitary and hypothalamus reported in patients after TBI (11). Antipituitary antibodies (APAs) were positive in 44.8% of patients 3 years after the TBI and were even reported 5 years after the initial diagnosis (12). The presence of APA is positively correlated with the development of pituitary insufficiency while their absence was associated with recovery in a 5-year prospective study (12).

## Case presentation

**Figure 1:**
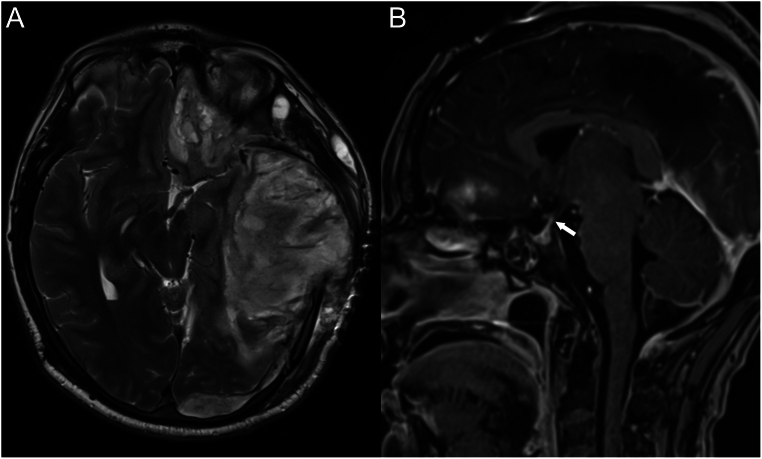
Axial T2 (A) depicts post traumatic contusions and vasogenic oedema in the right cerebral hemisphere and an occipital subdural haematoma resulting in mass effect and midline shift as well as extracranial cerebral herniation through the decompression craniectomy. Sagittal T1 3D Gd (B) shows stretching of the pituitary stalk due to the mass effect.

A 40-year-old male with no prior medical history suffered 8 years prior to presentation a severe TBI resulting in subdural hematoma with mass effect and uncal herniation requiring decompression hemicraniectomy (Fig. 1). The intervention was complicated by an epidural hematoma, subarachnoid haemorrhage and a large temporal intraparenchymal hematoma. The patient developed right motor hemi-syndrome, global aphasia, cognitive impairment, right hemi-negligence, right homonymous hemianopia and secondary epilepsy. The patient has undergone daily electrolyte testing following the TBI for 2 weeks, then weekly and yearly. None of these tests showed abnormalities of sodium. Additionally, the intensive care records do not mention polyuria.

Following the initial discharge, the patient had no emergency department (ED) or inpatient visits mentioned in his records. During the period since discharge, his caretakers did not report any symptoms such as polyuria or polydipsia. Seven years later, he visited the ED multiple times for one episode of seizure and frequent falls resulting in head trauma with mild TBI. A CT ruled out intracranial bleeding. Lastly, he showed behavioural changes in the last 3 months. He displayed physical aggressiveness towards anyone interfering with his increased water intake compatible with polydipsia and polyuria exceeding 5L a day (the patient has been seen numerous times drinking water directly out of sink taps). Sodium levels were normal as always and the head CT scan did not show any new abnormalities. He was admitted to the internal medicine ward for inpatient management of his behavioural disorder.

The initial hypothesis was primary psychogenic polydipsia. To prevent falls and wandering, physical restraints were used at night, which prevented the patient from drinking water in large quantities.

## Investigation

**Figure 2:**
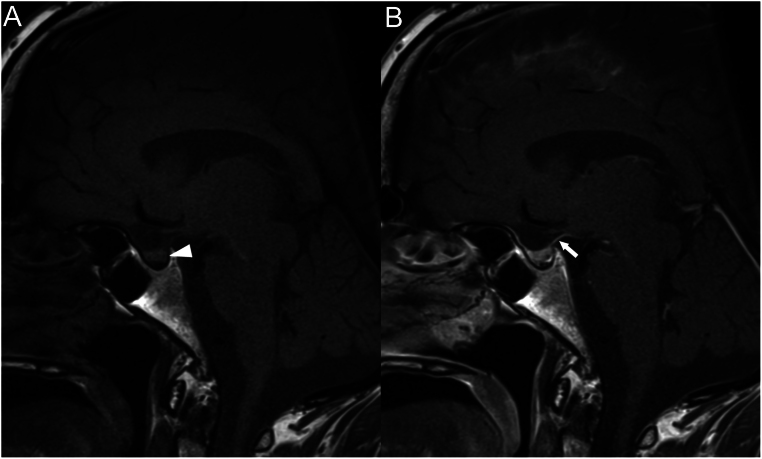
Sagittal non contrast (A) and post gadolinium (B) T1WI (A) shows absence of the spontaneous hyperintensity of the posterior hypophysis (arrowhead in A) and a very thin pituitary stalk (arrow in A), suggestive of post-traumatic changes.

The morning after, blood tests revealed a sodium (Na) of 149 mmol/L. Given the context, the suspicion of AVP-D was heightened. A pituitary magnetic resonance imaging (MRI) was ordered to confirm an absent or diminished posterior pituitary bright spot on T1WI (Fig. 2).

Carrying out the water restriction trial was challenging because it exacerbated the behavioural disorder, urinary catheterisation was unfeasible (risk of auto-extraction with secondary trauma) and the cognitive impairment rendered urinating by order impossible. We used levomepromazine as sedative and physical restraints to protect the staff. In order to avoid waking up the patient and aggravating his suffering we decided to skip the time points (Table 1). The morning urine sample yielded results compatible with AVP-D (hyponatremia and urine osmolality under 300 mmol/L).

**Table 1:** Laboratory results in each day of hospital stay. The results for day 2 and day 4 are influenced by night time water restriction trials.

Parameters	Initial results					Desmopressin 100 μg q.d.				
	Day 0	Day 1	Day 2*	Day 3**	Day 4*	Day 5	Day 6	Day 7	Day 9	Day 11
Sodium, mmol/L	143	149	154	143	147	140	141	141	143	143
Potassium, mmol/L	3.6	3.7	3.8	3.5	3.7	3.7	3.3	3.2	3.9	3.6
Glucose, mmol/L			5.8							
Serum osmolality (calculated), mOsm/kg			313							
Urine gravity			1.009		1.005					
Urine sodium			34		36					
Urine potassium			11		14					
Urine osmolality (calculated), mOsm/kg			152		169					

*water restriction; **pituitary MRI.

## Treatment

Desmopressin 100 μg once a day was initiated as treatment and as a diagnostic test. Consequently, blood tests showed a normal sodium level, and AVP-D was formally diagnosed based on the positive serum sodium response to the desmopressin correlated with the MRI findings. Estimating the response via urine osmolarity was not realised as collecting urine was impossible without restraints. The behavioural symptoms also resolved and the patient was discharged.

## Outcome and follow-up

The 2-month follow-up showed unchanged physical findings, a sodium level of 143mmol/L and a calculated osmolality of 291 mOsm/kg a urinary sodium level of 31 mmol/L and 134 mOsm/kg respectively. Clinically, the patient did not exhibit signs of hypothyroidism or hypogonadism provided that history could not be obtained. A screening for anterior hypopituitarism deficiency was considered positive because of low IGF-1 at 62 µg/L and low FSH (0.8 mUI/mL). The LH was normal (1.9 mIU/mL) and prolactin was mildly elevated (20.6 mIU/mL). The TSH was normal while the T4 was unfortunately not available. An ACTH stimulation with a conventional-dose (250 µg tétracosactide) short test showed an intact hypothalamic-pituitary-adrenal axis: the basal was 518 nmol/L and 755 nmol/L at 1 h.

Two months later the patient had a fall with head trauma complicated intra-parenchymal bleeding in the left frontal lobe, parafalcine and left convexity subdural hematomas and diffuse subarachnoid haemorrhage with an unfavourable outcome leading to his death.

## Discussion

Clinically, PTHP presents with symptoms specific to the deficient hormone. However, the nonspecific nature of symptoms such as cognitive impairment, neuropsychiatric symptoms and fatigue are often attributed to a post-concussion syndrome (13) thus rendering the diagnosis challenging. AVP-D should be considered in any patient with dilute urine output (14). In patients with dilute urine and elevated serum sodium (>147 mmol/L), vasopressin challenge testing should be performed. In patients with normal serum sodium, water deprivation testing can be used instead (15). In adults, AVP-D is characterized by polydipsia exceeding 3 L a day and polyuria exceeding 40–50 mL/kg/24 h (16). Although the water deprivation test is regarded as the gold standard for differentiating between AVP-D, AVP-R (arginine-vasopressine resistance), or primary polydipsia, copeptin the C-terminal peptide of the AVP pro-hormone can be used as a measure surrogate (16). In our case, the copeptin measurement was not available.

MRI of the pituitary gland, along with antibody tests and, if necessary, a biopsy, can help confirm the diagnosis of AVP-D (14). MRI of the pituitary gland is recommended for patients with suspected AVP-D to help distinguish it from primary polydipsia and evaluate for potential underlying causes such as tumours, or autoimmune processes (14). The posterior pituitary normally appears bright on T1-weighted MRI scans without contrast (gadolinium). Loss of this bright spot can indicate AVP-D. However, the bright spot can also be absent in patients with polydipsia or older adults without AVP-D. Thickening of the pituitary stalk or infundibulum greater than 3 mm is a nonspecific finding that has been associated with several conditions including autoimmune disorders, malignancy, and other entities such lymphocytic hypophysitis (14).

In our case, although not dedicated, the MRI following the initial TBI revealed an intact but stretched pituitary stalk (Fig. 1) while the second MRI did confirm a thin stalk and loss of the pituitary bright spot on the MRI (Fig. 2). We interpret this as a late destructive process giving the absence of these modifications on an initial post-TBI MRI.

The development of anterior pituitary insufficiency also indicates that the patient’s AVP-D was not solely due to the acute TBI. Autoimmunity may also have contributed, but antipituitary antibodies testing was not carried out. Repeat mild TBIs from falls may have further damaged the pituitary. The cumulative effects of ongoing neuroinflammation, vascular impairment, and autoimmunity likely combined to cause gradual pituitary failure manifesting as delayed AVP-D and the suspected anterior pituitary insufficiency.

It is also possible that the patient had ongoing mild AVP-D that went initially unnoticed due to cognitive impairment. If this was the case, the multiple falls in the year prior to admission may have exacerbated his pre-existing AVP-D. Another possibility is that the patient did sustain unwitnessed or unreported falls with mild TBI, which might have led to PTHP in an incremental fashion. AVP-D after mild TBI has been reported in the literature (17).

The British Neurotrauma Group guidance states in their paper from 2017 that acute testing for pituitary disfunction is not recommended for the purpose of detecting post-TBI hypopituitarism. However, in case of clinical suspicion of cortisol insufficiency, an empirical replacement therapy with hydrocortisone should be started. AVP-D should be considered at an early stage in patients with TBI displaying hypernatremia and hypotonic polyuria. Following discharge, each patient who required an admission for more than 48 h or who has ongoing symptoms should undergo screening at 3–6 months to rule out pituitary dysfunction. Finally, patients with symptoms of pituitary symptoms should also be screened for depression (18).

Conversely, Gilis-Januszkewska *et al.* proposed screening guidelines for hypopituitarism after TBI. They recommend screening all moderate TBI patients, severe TBI patients with good life expectancy, and mild TBI patients with skull fractures, elderly patients, patients with polyuria/polydipsia, or those sustaining repeated head injuries. In the first week, they suggest screening for corticotropin deficiency. Within 12 months, they recommend screening for deficiencies in TSH, LH, FSH, as well as AVP-D (19).

This case demonstrates that AVP-D can present many years after TBI, likely due to progressive damage to the pituitary gland. A high index of suspicion is needed to diagnose delayed onset hypopituitarism in patients with a history of TBI as the symptoms are non-specific. Periodic screening for anterior and posterior pituitary dysfunction should be considered in patients with TBI.

## Declaration of interest

There is no conflict of interest that could be perceived as prejudicing the impartiality of the study reported.

## Funding

This work did not receive any specific grant from any funding agency in the public, commercial or not-for-profit sector.

## Patient consent

Every effort was made to contact the next of kin of the deceased patient to obtain consent but was unsuccessful.

## Author contribution statement

ST was the physician in charge of the patient, did the literature review and wrote the initial manuscript. JB analysed, chose the MRI images, and proofread the article. KG was the consulting endocrinologist during in-patient setting, supervised the writing and submission process.
